# Highly Sensitive Detection of CA 125 Protein with the Use of an n-Type Nanowire Biosensor

**DOI:** 10.3390/bios10120210

**Published:** 2020-12-18

**Authors:** Kristina A. Malsagova, Tatyana O. Pleshakova, Rafael A. Galiullin, Andrey F. Kozlov, Ivan D. Shumov, Vladimir P. Popov, Fedor V. Tikhonenko, Alexander V. Glukhov, Vadim S. Ziborov, Oleg F. Petrov, Vladimir E. Fortov, Alexander I. Archakov, Yuri D. Ivanov

**Affiliations:** 1 Laboratory of nanotechnology, Institute of Biomedical Chemistry, 119121 Moscow, Russia; t.pleshakova1@gmail.com (T.O.P.); rafael.anvarovich@gmail.com (R.A.G.); afkozlow@mail.ru (A.F.K.); shum230988@mail.ru (I.D.S.); ziborov.vs@yandex.ru (V.S.Z.); alexander.archakov@ibmc.msk.ru (A.I.A.); yurii.ivanov.nata@gmail.com (Y.D.I.); 2 Rzhanov Institute of Semiconductor Physics, Siberian Branch of Russian Academy of Sciences, 630090 Novosibirsk, Russia; popov@isp.nsc.ru (V.P.P.); ifp@isp.nsc.ru (F.V.T.); 3 JSC Novosibirsk Plant of Semiconductor Devices with OKB, 630082 Novosibirsk, Russia; gluhov@nzpp.ru; 4 Joint Institute for High Temperatures of Russian Academy of Sciences, 125412 Moscow, Russia; ofpetrov@ihed.ras.ru (O.F.P.); fortov@ihed.ras.ru (V.E.F.)

**Keywords:** ovarian cancer, nanowire biosensor, nanowire, silicon-on-insulator, CA 125, antibodies

## Abstract

The detection of CA 125 protein in a solution using a silicon-on-insulator (SOI)-nanowire biosensor with n-type chip has been experimentally demonstrated. The surface of nanowires was modified by covalent immobilization of antibodies against CA 125 in order to provide the biospecificity of the target protein detection. We have demonstrated that the biosensor signal, which results from the biospecific interaction between CA 125 and the covalently immobilized antibodies, increases with the increase in the protein concentration. At that, the minimum concentration, at which the target protein was detectable with the SOI-nanowire biosensor, amounted to 1.5 × 10^−16^ M.

## 1. Introduction

The effective treatment of most pathologies, including cancer, depends on the early revelation of a pathological process [1]. The identification of target biomarkers, associated with the early-stage (asymptomatic) development of a disease, is a starting point for choosing an appropriate and effective treatment. Most of the protein markers are present in the blood at low (<10^−13^ M) or ultra-low (<10^−15^ M) concentrations. The blood concentration of cancer biomarkers at the early stage of an oncological disease is at the level of 10^−15^ M (that is, at the femtomolar level), as was emphasized by Rissin et al. [2]. The early revelation of cancer in human, accordingly, requires the development of novel methods, which allow for one to detect cancer biomarkers with, at least, femtomolar concentration sensitivity. The application possibilities of standard immunohistochemical, radioimmunoassay (RIA)-based, enzyme-linked immunosorbent assay (ELISA)-based methods, etc. (which are commonly employed in clinical diagnostics for the detection of protein markers), are limited due to: (1) their low (10^−14^ M to 10^−7^ M) concentration sensitivity and (2) the need to use enzyme and fluorescent labels.

The use of nanowire biosensors gives new opportunities for biomedical research, as well as for clinical practice in the future. One of the key advantages of this type of biosensors consists in the possibility of direct label-free detection of a target protein in real-time with high (<10^−15^ M) concentration sensitivity [3]. The operation of a nanowire biosensor is based on the registration of a modulation of the electric current through the nanowire sensor elements upon adsorption of target protein molecules onto the surface of the sensor elements. The surface-adsorbed molecules act as a virtual gate, and the nanowire structure itself with ohmic contacts on its ends acts as a nanoscale field-effect transistor (FET) [4]. The high sensitivity of the nanowire sensor element is determined by its high surface-to-volume ratio [5]. The theoretical detection limit, which is attainable with a nanowire biosensor, can reach the level of a single molecule per sensor element [6]. In this way, F. Patolsky et al. [7] reported that the use of nanowire biosensors allows for the detection of viruses with the sensitivity at the single-particle level. Regarding biological macromolecules, the femtomolar detection limit was experimentally attained for DNA [8,9]; for proteins, an even lower (subfemtomolar) detection limit was attained [3].

The surface of nanowire sensor elements is functionalized with biospecific probe molecules (molecular probes) in order to provide the biospecificity of the detection of target protein markers of diseases. The formation of an array, containing multiple nanowires on a single chip, with subsequent functionalization of the nanowires with molecular probes against various types of target biomolecules represents another important advantage of the nanowire biosensors, since this allows for one to conduct multiplexed detection of target proteins in one sample. Thus, nanowire biosensors combine the following advantages: (1) highly sensitive label-free detection of target proteins and the (2) rapid simultaneous express analysis of a wide range of target proteins.

CA 125 protein represents a marker, which is associated with the development of malignant tumors (ovarian cancer, uterine cancer, endometrial cancer, breast cancer, etc.), benign tumors (endometriosis, pleurisy, etc.), and inflammatory diseases [10]. The discovery of this antigen has become an important step on the way to the development of a biochemical approach to the non-invasive diagnosis and monitoring of ovarian cancer. The use of CA 125 as a marker of ovarian cancer was suggested in 1983 and, since this time, it has been considered as the benchmark for monitoring ovarian cancer patients [11]. CA 125 represents a glycoprotein epitope of a mucin with high molecular weight [10]. One of the main causes of errors, which occurs during biomarker detection, is their biological variability [12]. The use of CA 125 is limited by the low sensitivity of the marker (<50%) for the initial stage of the disease and its poor specificity, especially in young women. However, the results of twenty-three randomized large-scale screening research studies on 250,000 women suggest the benefits of screening that is based on CA 125 evaluation, in order to early detect ovarian cancer in menopause-aged women, as well as in women with familial clustering of ovarian cancer [13].

Herein, antibodies against CA 125 have been employed as molecular probes for the functionalization of an n-type nanowire biosensor chip. This chip was fabricated on the basis of silicon-on-insulator (SOI) structure employing complementary metal–oxide–semiconductor (CMOS)-compatible technology. In contrast to our previous studies [14,15,16,17,18], before the surface functionalization, the sensor chips were treated with glow discharge plasma instead of ozone treatment. The antibody-functionalized chips have been used for the highly sensitive detection of high molecular weight glycoprotein CA 125—a protein marker of ovarian cancer—in purified buffer solution. The experimentally attained concentration detection limit of CA 125 was ~10^−16^ M. Because the early diagnosis of oncological pathologies requires the use of highly sensitive detection methods, which allows for attaining a 10^−15^ M concentration detection limit [2], our nanowire biosensor represents an attractive tool for the rapid express analysis of protein markers of oncological diseases.

## 2. Materials and Methods

### 2.1. Chemicals

3,3′-dithiobis (sulfosuccinimidyl propionate) (DTSSP cross-linker) was purchased from Pierce (Waltham, MA, USA). Potassium phosphate monobasic (KH_2_PO_4_) and 3-aminopropyltriethoxysilane (APTES) were purchased from Sigma–Aldrich (St. Louis, MO, USA). Methanol (CH_3_OH) was purchased from Sigma (St. Louis, MO, USA). Hydrofluoric acid (HF) was purchased from Reakhim (Moscow, Russia). Deionized water was obtained while using a Milli-Q system (Millipore, Molsheim, France).

### 2.2. Proteins

Monoclonal antibodies against CA125 (clone 13F4, isotype IgG1) were purchased from USBio (Salem, MA, USA). The recombinant СА125 protein (molecular weight 110 kDa; 10^−6^ M stock solution in potassium phosphate buffer) was purchased from R&D Systems (Minneapolis, MN, USA).

Antibodies against Bcl-2 protein were purchased from Biorbyt, Ltd. (Cambridge, UK).

### 2.3. Fabrication of Nanowire Sensors

**Figure 1:**
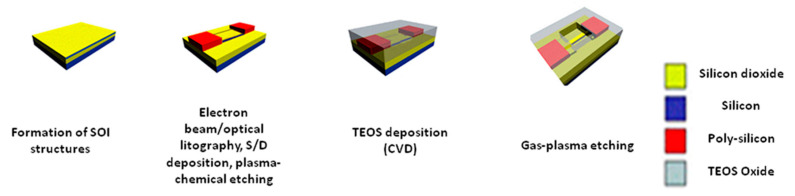
Schematic representation of silicon-on-insulator-NW (SOI-NW) sensor fabrication.

The fabrication and characteristics of the SOI-nanowire sensor chips (SOI-NW chips) are described in detail elsewhere [15,16]. The process of silicon-on-insulator (SOI) nanowire chips fabrication, as schematically shown in Figure 1, comprised of the following steps: the production of initial SOI structures with a cut-off Si layer thickness of 500–600 nm while using hydrogen exfoliation technology; thinning of the SOI layer to nanometer dimensions by a sequential cycle of operations-thermal oxidation; removal of sacrificial oxide in HF solution; lateral structuring of the SOI layers using optical or electron lithography to form nanowire structures with contact areas; the formation of ohmic contacts to nanometer thick SOI layer by thickening the SOI in the contact areas by a poly-Si layer deposition and subsequent doping; lateral structuring of SOI layers while using electronic lithography and gas-plasma chemical etching, which allows for one to form a nanometer-size active element; metallization and contact wiring; and, finally, crystal cutting.

**Figure 2:**
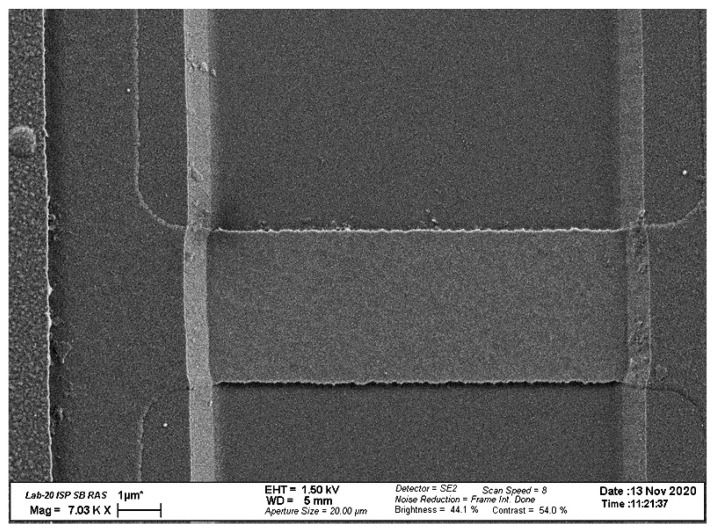
Typical SEM image of a single nanowire sensor element.

In our biosensor, SOI-NW chips with n-type conductance were employed. The thickness of the cut-off silicon layer was 32 nm and the buried oxide (BOX) thickness was 300 nm. The width of the nanowire sensor elements was *w* = 3 μm, while their thickness and length were *t* = 32 nm and *l* = 10 μm, respectively. The number of nanowires on the crystal was 12. Figure 2 displays the typical SEM image of a single nanowire sensor element.

### 2.4. Modification of the Surface of the SOI-Nanowire Chip

The surface of the SOI-NW chips was first treated chemically, in order to remove the organic contaminants and the natural oxide from the sensor surface, with isopropanol, HF, and CH_3_OH similarly to the procedure that was described in our previous papers [14,15,16,17,18]. After that, the chips were treated with glow discharge plasma in a homemade apparatus that was developed in JIHT RAS, in order to form OH groups on the sensor surface, and then the chip was treated in APTES vapors, according to [17,18].

### 2.5. Covalent Immobilization of Molecular Probes

The molecular probes (antibodies against CA 125 and against Bcl-2) were covalently immobilized onto the chemically modified surface of the nanowires with the use of the DTSSP crosslinker. For this purpose, 3-nL microdrops of 0.8 μM solutions of antibodies in potassium phosphate (KP) buffer (50 mm, pH 7.4) were precisely dispensed onto the surface of individual DTSSP-activated nanowires with a Piezorray micro-arraying system (PerkinElmer, Inc., Waltham, MA, USA). The solutions were incubated on the surface of the nanowires for 30 min. at 15 °C and 80% humidity. After that, the surface of the chip was washed with deionized water for 30 min.

### 2.6. Preparation of CA 125 Solutions in Buffer

CA 125 solutions with concentrations that ranged from 10^−18^ M to 10^−15^ M were prepared from the initial stock solution of the protein (1 μM in 50 mM KP, pH 7.4) by sequential tenfold dilution with 1 mM KP buffer (pH 7.4). On each dilution step, the protein solution was incubated in a shaker at 10 °C for 30 min. The so-prepared protein solutions were then immediately used in the biosensor measurements.

### 2.7. Electrical Measurements

The nanowire biosensor setup is described in detail in [19]. The electrical measurements were performed with a Keithley Model 6487 picoampermeter (Keithley, Solon, USA). During the measurements, the substrate of the SOI structures was used as the control electrode (transistor gate). In the course of the experiments, the dependence of source-drain current on gate voltage *I_ds_*(*V_g_*) at *V_g_* from 0 to 100 V and *V_ds_* = 0.15 V was obtained for the SOI-NW chip. In order to detect the target protein, 150 μL of CA 125 solution in 1 mM KP buffer (pH 7.4) was added into the measuring cell containing 300 μL of buffer solution. The time dependencies of the current *I_ds_*(*t*) were recorded at *V_g_* = +50 V and *V_ds_* = 0.15 V. We used an additional Pt electrode, which was immersed into the solution in the measuring cell (similar to [3]), in order to increase the time stability of the nanowire sensors with a thin nanoconductor.

The biosensor included a 500-μL measuring cell, and the sensor chip with an array of nanowires served as the cell bottom. The diameter of the sensitive area was 2 mm. The solution in the cell was stirred at 3000 rpm.

## 3. Results

The detection of CA 125 was carried out in the measuring cell of the nanowire biosensor, while using a SOI-NW chip bearing an array of twelve 3-μm-thick n-type nanowires. The nanowires were functionalized by covalent immobilization of antibodies against CA 125 onto their surface in order to provide biospecificity of the CA 125 detection (as described in Materials and Methods). In order to account for the non-specific signal, a pair of nanowires of the same thickness (3 μm) was functionalized with antibodies against Bcl-2. The signal from these sensors was taken into account in order to calculate the resulting differential signal.

The sensograms were recorded before and after the addition of the CA 125 solutions in 1 mM KP buffer (pH 7.4) with the protein concentration ranging from 10^−18^ M to 10^−14^ M into the measuring cell of the biosensor.

**Figure 3:**
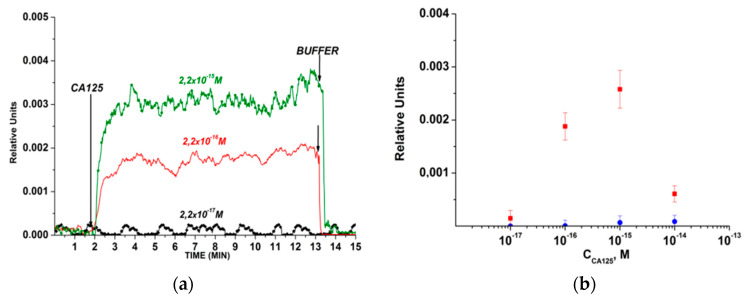
The results obtained upon the detection of СА125 protein in buffer solution while using an n-type SOI-NW chip with covalently immobilized antibodies: (**а**) typical sensograms obtained upon analysis of solutions with various concentrations of the target protein; (**b**) dependencies of the level of the biosensor signal on the concentration of CA 125 in buffer solution. The number of technical replicates was *n* = 3. Circles (●) and squares (■) indicate the average value of the signal level before and after the addition of the protein solution, respectively. The experimental conditions: 1 mM potassium phosphate (KP) buffer, pH 7.4, *V_g_* = +50 V; *V_ds_* = 0.15 V. The total volume of the solution in the cell was 450 μL. Arrows indicate the addition of the CA 125 solution (with concentrations from 2 × 10^−18^ to 2 × 10^−14^ M, as indicated in the Figure) and the wash with pure KP buffer.

Figure 3 displays typical sensograms that were recorded before and after the addition of CA 125 solutions into the measuring cell. The sensogram curves indicate that the addition of CA 125 solution leads to an increase in the signal from the nanowires with immobilized antibodies, owing to the binding of the target analyte molecules to their surface (Figure 3). Figure 3 shows that, in the case of using the SOI-NW chip with immobilized antibodies, the biosensor signal was clearly distinguishable until reaching the target protein concentration of 1.5 × 10^−16^ M.

The control experiments were carried out in order to determine the non-specific influence of the protein-free buffer on the biosensor signal. In the control experiments, upon the addition of the analyte-free working buffer into the measuring cell, either no response from the nanowires was observed or this response was no greater than 1 to 2% of the baseline signal level.

Moreover, a decrease in the response signal from the nanowires with decreasing the target protein concentration from 10^−15^ M to 10^−17^ M was observed.

These results allow for us to make a conclusion regarding the presence of the biospecific interaction between the molecular probes, immobilized on the SOI-NW chip surface, and the target protein molecules that are captured from the analyzed solution onto this surface.

It should be emphasized that the substitution of CA 125 solution with a protein-free buffer solution led to a decrease in the signal from the nanowires; in other words, it caused the dissociation of the СА 125/(antibodies against СА 125) complexes due to the shift in the biospecific interaction’s equilibrium. This fact indicates the possibility of repeated use of the SOI-NW sensor chip for the detection of CA 125.

Upon increasing the target protein concentration to 2.2 × 10^−14^ M and higher values, no difference between the signal, which is received from the control nanowires, and that from working nanowires, was observed. This fact can be explained by the high degree of non-specific binding of target molecules to the surface of the control nanowire. A large number of molecules can lead to the oligomerization of the target protein and, consequently, to a change in the physicochemical parameters of the interaction of the target analyte molecules with the sensor surface—for instance, to a change in the efficiency of the protein adsorption onto the surface of the control nanowire.

The results obtained herein indicate that the immobilized molecular probes retain their affinity properties, and this allows for the biospecific capturing of the target protein onto the sensor surface. In our experiments, the lowest concentration of the target CA 125 protein, which was detectable with the antibody-functionalized SOI-NW chip, was 2.2 × 10^−16^ M.

In our present study, we have demonstrated the possibility of the nanowire biosensor-based detection of CA 125 oncomarker, employing purified solutions of a commercial CA 125 preparation in buffer and antibody-functionalized nanowire sensor chips, attaining a 10^−16^ M concentration detection limit. It should be emphasized that, in the case of viral infections (such as HCV infection [17]), their nucleic acid markers can be detected while using a polymerase chain reaction (PCR)-based assay. Moreover, nucleic acid molecules bear a large amount of negative charge—in contrast to the case with the majority of proteins—and, hence, represent objects that are much more easily detectable with a nanowire biosensor. In contrast, the diagnosis of ovarian cancer (as well as other oncological diseases) in human requires the detection of protein markers, and this is the approach that we develop in our present study. It is known that the early diagnosis of oncological pathologies requires the use of highly sensitive detection methods, which allow for one to attain a 10^−15^ M concentration detection limit [2]. In this respect, nanowire biosensor allows for one to overcome the 10^−15^ M sensitivity threshold, thus representing a quite attractive tool for the rapid detection of protein markers of oncological diseases. Moreover, the 10^−16^ M detection limit, attained with the use of a nanowire biosensor in our present study, is not an ultimate point, and it can be further shifted down. One of the ways to lower the detection limit is the use of a scheme involving a microwave generator, as was reported in one of our previous papers [17]. In addition, decreasing the width of the nanowire sensor elements, providing higher surface-to-volume ratio [5], is another way for further increasing the sensitivity of nanowire biosensors. In principle, decreasing the nanowire width can allow for the single-charge sensitivity of the biosensor [5], which means the possibility to perform nanowire-based detection of charged biomolecules with single-molecule sensitivity, and this is what we will aim for in future research.

Moreover, our nanowire sensor chips are fabricated while using a CMOS-compatible technology, and this is another advantage of the biosensor proposed herein, as it allows for the transition to large-scale production, providing low cost of the sensor chips—which is required for the clinical screening applications of the approach being developed.

## 4. Conclusions

Herein, the highly sensitive detection of cancer-associated protein marker CA 125 in buffer solution (at pH 7.4) with a nanowire biosensor has been experimentally demonstrated. Silicon-on-insulator (SOI) structures, which were fabricated using top-down technology, were used as sensor chips. For the functionalization of the sensor surface, antibodies against CA 125 were used as biospecific molecular probes. The use of antibody-functionalized SOI-NW chips has allowed us to experimentally attain the concentration limit of CA 125 detection at the level of 2.2 × 10^−16^ M.

The results obtained herein indicate that the nanowire biosensor represents a prototype of a medical diagnostic device, which can be employed for the revelation of cancer. Moreover, because our nanowire biosensor includes a sensor chip bearing an array of 12 nanowires, its application will allow for one to perform the simultaneous selective early diagnosis of a number of common and socially significant diseases in one test, which seems to be promising for screening applications in medical diagnostics.
